# Web-Based Recruiting for Health Research Using a Social Networking Site: An Exploratory Study

**DOI:** 10.2196/jmir.1978

**Published:** 2012-02-01

**Authors:** Yeshe Fenner, Suzanne M Garland, Elya E Moore, Yasmin Jayasinghe, Ashley Fletcher, Sepehr N Tabrizi, Bharathy Gunasekaran, John D Wark

**Affiliations:** ^1^Department of Microbiology and Infectious DiseasesRoyal Women’s HospitalParkvilleAustralia; ^2^Infection and Immunity ThemeMurdoch Childrens Research InstituteParkvilleAustralia; ^3^Department of Obstetrics and GynaecologyUniversity of MelbourneParkvilleAustralia; ^4^Department of MicrobiologyRoyal Children’s HospitalParkvilleAustralia; ^5^Department of GynaecologyRoyal Children’s HospitalParkvilleAustralia; ^6^Melbourne Medical SchoolUniversity of MelbourneParkvilleAustralia; ^7^Department of MedicineRoyal Melbourne HospitalUniversity of MelbourneParkvilleAustralia; ^8^Bone and Mineral ServiceRoyal Melbourne HospitalParkvilleAustralia

**Keywords:** Advertising, research subject recruitment, women’s health, Facebook

## Abstract

**Background:**

Recruitment of young people for health research by traditional methods has become more expensive and challenging over recent decades. The Internet presents an opportunity for innovative recruitment modalities.

**Objective:**

To assess the feasibility of recruiting young females using targeted advertising on the social networking site Facebook.

**Methods:**

We placed an advertisement on Facebook from May to September 2010, inviting 16- to 25-year-old females from Victoria, Australia, to participate in a health study. Those who clicked on the advertisement were redirected to the study website and were able to express interest by submitting their contact details online. They were contacted by a researcher who assessed eligibility and invited them to complete a health-related survey, which they could do confidentially and securely either at the study site or remotely online.

**Results:**

A total of 551 females responded to the advertisement, of whom 426 agreed to participate, with 278 completing the survey (139 at the study site and 139 remotely). Respondents’ age distribution was representative of the target population, while 18- to 25-year-olds were more likely to be enrolled in the study and complete the survey than 16- to 17-year-olds (prevalence ratio = 1.37, 95% confidence interval 1.05–1.78, *P* = .02). The broad geographic distribution (major city, inner regional, and outer regional/remote) and socioeconomic profile of participants matched the target population. Predictors of participation were older age, higher education level, and higher body mass index. Average cost in advertising fees per compliant participant was US $20, making this highly cost effective.

**Conclusions:**

Results demonstrate the potential of using modern information and communication technologies to engage young women in health research and penetrate into nonurban communities. The success of this method has implications for future medical and population research in this and other demographics.

## Introduction

Recruiting participants into health studies has become increasingly challenging. Traditional strategies, such as school-based recruitment, random digit dialing, systematic door knocking, and media advertising campaigns, have limitations including low participation rates [1], decreasing frequency of fixed household telephone line connections [2], and high costs [3,4]. Young people in particular are underrepresented in medical and population-based studies, as they are highly mobile, and recruitment and retention are difficult [5]. Modern social and technological changes have implications for research involving young people. A recent survey reported that 93% of 12- to 17-year-olds and 89% of 18- to 24-year-olds in the United States had access to the Internet [6], and the majority of these young people used the Internet daily [7]. An even more recent phenomenon is the dramatic rise in popularity of online social networking sites. The most popular social networking site is Facebook, with an estimated 800 million active users worldwide, of whom 50% will log on to Facebook in any given day [8]. While Facebook is already well established in developed nations, it is truly a global phenomenon, with the biggest growth in usage occurring in developing countries [9]. In Australia, use of social networking sites is the number one online activity for 16- to 29-year-olds, with 83% using them on a regular basis [10] and 93% of social networking site users being Facebook members [11]. Because social interactions between young people commonly occur via the Internet, social networking sites offer a promising new way to recruit participants, particularly young people, into medical research.

Web-based recruitment methods have been reported previously, including paid advertising and links on websites and online discussion boards [12-14]. We are aware of only a few publications describing health studies that used paid Facebook advertising to recruit participants [15-22]*.* Most of these studies grouped Facebook advertising with other online free and paid advertising strategies and did not compare the demographics of an exclusively Facebook*-*recruited sample with the target population. For instance, one study compared three methods for inviting young adult smokers to complete a survey [20]: (1) advertisements on the free online classifieds page Craigslist.org, (2) other Internet advertisements (including Facebook, MySpace, other social networking sites, Google, and file sharing and entertainment streaming websites), and (3) invitations to members of Internet market research panels. Method 2, which attracted younger participants and more males than the other methods, yielded the most completed surveys overall, while methods 1 and 3 were more cost effective and attracted participants more likely to complete the survey. However, the authors did not report demographic characteristics by Internet advertisement type. Another study that used Facebook advertisements in 2005 to invite US college students to complete a survey about prescription opioid misuse found that males and white students were more likely to respond to the advertisement; however, this may be consistent with the demographic profile of people who misuse prescription opioids [19]. At the time of that study, Facebook was far less popular than today with an audience of about 2.5 million users and open only to students with an educational email address (ie, extension .edu), making it difficult to generalize their results. Other studies have recruited participants by creating Facebook group pages and employing chain referral, or snowball sampling, to exploit group and friendship connections between Facebook users to obtain a convenience sample [15,23-25]. While this technique may be efficient and cost effective, it has limited potential to attract a representative sample, due to the reliance on social connections.

Our study differs from these previous studies in the following key ways: (1) recruitment at a time when the vast majority of the target population are regular Facebook users, thus giving the sampling modality a potentially broad reach, (2) systematic monitoring of each stage of recruitment including the display of, and response to, the advertisement, and navigation through our website (and as a function of age and regional group), and (3) assessment of the representativeness of a sample recruited exclusively using targeted Facebook advertising.

Our objectives were to assess (1) the feasibility of obtaining a representative sample of young females, using the Facebook targeted advertising system, which presents advertisements to users based on a selection of prespecified characteristics including location, age, and gender, and (2) young females’ knowledge of and attitudes toward health issues, and participation in health and medical research. This was an exploratory study and we had no a priori hypotheses regarding these objectives.

## Methods

### Study Design

Inclusion criteria for participation in this cross-sectional study were (1) female, (2) 16–25 years old, (3) living in the Australian state of Victoria, and (4) willing to complete a health survey. The survey asked questions about demographic data, sexual and reproductive health, and willingness to participate in a larger health study*.* Exclusion criteria were perceived inability to give informed consent or complete the questionnaire due to inadequate understanding of the purpose and procedures of the study. We selected a target sample size of 200 as a reasonable number of participants to enroll within our budget and time frame.

### Procedures

**Figure 1:**
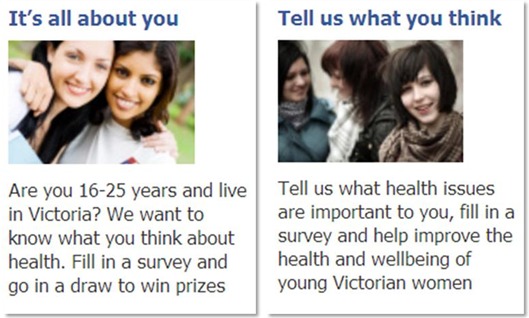
Examples of Facebook advertisements.

Facebook advertisements were displayed to Facebook users whose profiles matched our inclusion criteria: (1) between the ages of 16 and 25 years, inclusive, (2) female, and (3) located in Victoria, Australia. Age and gender are based on the information listed in the user’s Facebook profile (age and gender are required by Facebook for all personal accounts), while location is based on the Internet protocol address or the address listed on the user’s profile [26]. At the time of creating these Facebook advertisements (April 2010), city-level, but not state-level, targeting was available for Australia. Therefore, we used city-level and geographic radius targeting [26] to target advertisements to people located within 50-mile radii of 17 cities throughout the state of Victoria. We used the largest possible radii and selected cities to maximize coverage of the state of Victoria. The Facebook advertisements comprised (1) one of several short titles (eg, “It’s all about you,” or “Tell us what you think”), (2) an image (eg, photos of young women of various ethnic backgrounds engaging in exercise or social activities), and (3) main text up to 135 characters in length (eg, “Are you 16–25 years and live in Victoria? We want to know what you think about health. Fill in a survey and go in a draw to win prizes,” or “Tell us what health issues are important to you, fill in a survey and help improve the health and wellbeing of young Victorian women”) (Figure 1).

Facebook gives advertisers the choice of being charged each time the advertisement is clicked (cost-per-click) or each time the advertisement is displayed a certain number of times (cost-per-thousand-impressions) [27]. We chose the cost-per-click option, as we were interested in people clicking through to our website. The advertiser also chooses a bid, which is the maximum the advertiser will pay for each click on the advertisement, in the cost-per-click model. From the available ad inventory, the Facebook advertising algorithm automatically selects the best advertisement to run based on advertisement performance and the cost-per-click or cost-per-thousand-impressions. Facebook advertisements compete with each other to appear in the ad space on the right-hand side of the webpage. For each advertisement, Facebook gives a suggested bid range, which is the range of bids currently winning the auction among similar advertisements being displayed to the targeted audience; a low bid makes it unlikely that the advertisement will be displayed [27]. Our bids ranged from US $0.70 to US $1.15 and fell within Facebook’s suggested bid range. Our daily budget (the maximum Facebook charge per day; once reached, Facebook stops running the advertisements for that day) ranged from US $20 in the first week of advertising up to US $90 in the final week of advertising, and was adjusted according to our desired recruitment rate.

Advertisements appeared on Facebook from May 19 to September 29, 2010. From May 19 to June 29, 2010, we conducted a single advertising campaign, targeting all female Victorian Facebook users aged 16–25 years. Subsequently, we used six separate advertising campaigns to target each combination of three age groups (16–17, 18–21, and 22–25 years) and two regions (urban and nonurban), to obtain more detailed demographic information and allow the advertising budget for each campaign to be adjusted, if needed, to try to yield a representative sample. For instance, if fewer advertisers were competing to advertise to female adolescents under 18 years of age, having one single advertising campaign for 16- to 25-year-olds may lead to our advertisements being displayed preferentially to under 18s, due to the nature of Facebook’s ad bidding process, without any way for us to control this. As above, we used city-level and geographic radius targeting parameters to target advertisements to people in urban and nonurban Victoria. For the urban advertising campaigns, advertisements were targeted at females located within 10 miles of Melbourne, the capital city of Victoria. For the nonurban advertising campaigns, advertisements were targeted at females located within 50 miles of 12 cities, an area that covered most of regional Victoria while excluding major cities.

When a Facebook user clicked on an advertisement, she was redirected to our secure study website (www.yfhi.org), containing study information (details about the objectives and procedures of the study, eligibility criteria, prize draw for completing the survey, and researchers’ backgrounds, affiliations, and contact information) and an expression-of-interest form to enable users to learn more about the study.

Potential participants who visited our study website could either send us their telephone and email contact information through a secured encrypted online system, or directly contact study personnel. Research staff assessed eligibility of all participants over the telephone after initial contact, explained the study, and assessed respondents’ competence to give informed consent (based on their ability to understand the purpose and procedures of the study and explain it in words). Those eligible and interested were invited to visit a study site in the suburb of North Melbourne, Victoria, to complete a health-related survey. The study site was an office suite located 2 km north of the city center of Melbourne, Victoria, in a medical precinct with several major hospitals situated nearby and readily accessible by public transport. Respondents who declined to visit the study site were then invited to complete the survey online remotely. The reason for initially inviting respondents to visit our study site was to assess the proportion of young females who would travel to participate, which informs the suitability of this sampling method for recruiting females into future studies requiring in-person contact. Respondents under 18 years of age underwent a mature minor assessment by a researcher following guidelines from the Medical Practitioners Board of Victoria, *Consent for T* *reatment and C* *onfidentiality in Y* *oung P* *eople* [28], on how to define a mature and competent young person. Briefly, the researcher assessed age, general maturity of speech, level of schooling, and ability to understand the nature and rationale of the research project and to explain it in words. Verbal consent was obtained from all participants by telephone. In addition, written consent was obtained from those participants visiting the study site.

Respondents were considered unreachable after no response to three missed telephone calls, plus an SMS message and/or email. Respondents who initially consented to participate were considered lost to follow-up if they were unreachable to schedule an appointment at the study site, or did not complete the online survey remotely after three reminder emails. Respondents who were initially consented into the study site group, but who subsequently were unable or unwilling to visit the study site, were given the option of completing the survey remotely.

Participants were offered AU $25 (AU $1 = US $1.05 in April 2011) compensation for their time and travel costs if they visited the study site (and up to AU $70 additional travel reimbursement if travelling from regional areas) or AU $15 compensation for their time for completing the survey remotely.

Participants who visited the study site were asked to complete the online survey at a computer in a private room. Those who participated remotely were emailed instructions for accessing the online survey. We used the online survey tool Survey Monkey (www.surveymonkey.com), with the enhanced security option of secure socket layers encryption, to administer the survey to participants. To further protect privacy, we masked participants’ Internet protocol addresses so that they were not stored in the survey results. To enable compliance monitoring, researchers provided participants with a unique study identification number to access the survey. The survey contained questions about demographic variables (date of birth, marital status, living arrangements, income, country of birth, education, employment status, indigenous status, ethnicity, and postal/zip code), height and weight, how they found out about the study, sexual history, experience and knowledge of sexually transmitted infections (*Chlamydia trachomatis* and human papillomavirus), and the acceptability of participating in a long-term research study, including answering sensitive questions and undergoing physical examinations (Multimedia Appendix 1).

### Statistical Analysis

Statistical analyses were performed using Stata version 11.1 (StatCorp LP, College Station, TX, USA). We used Australian Bureau of Statistics 2006 census data [29] and Victorian Population Health Survey 2008 data [30] to compare our cohort with the general population. Socioeconomic status was assigned using the Bureau’s Socio-Economic Indexes For Areas (SEIFA) and the 2006 Postal Area Index of Relative Socio-economic Advantage and Disadvantage, which is a continuum of advantage (high values) to disadvantage (low values) scores [31].

We compared sociodemographic characteristics (age group, geographic region, country of birth, indigenous status, socioeconomic level, and education level) and body mass index (BMI) in our sample with that of the general population using a Fisher exact test.

Prevalence ratios (PRs), 95% confidence intervals (CIs), and 2-sided *P* values were estimated using log-binomial regression [32]. When the log-binomial model failed to converge, we used a Poisson model with robust error variance as an approximation [32]. We estimated PRs of clicking on the advertisement, mutually adjusting for geographic region and age group, and PRs of submitting an expression of interest, stratifying by age group. Associations with visiting the study site to complete the survey, rather than completing it online remotely, were also estimated using PRs, mutually adjusting for age group, geographic region, country of birth, socioeconomic level, education level, and BMI.

In all analyses, we defined a 2-sided *P* value of <.05 as statistically significant. Data were treated as missing if no response was given or “don’t know” was selected.

### Ethical Considerations

We obtained ethical approval for the study through the Human Research and Ethics Committees at the Royal Women’s Hospital, Melbourne, Australia, and adhered to the *National Statement on Ethical Conduct in Human Research* [33], which was developed to protect the interests of people who participate in research studies.

The confidentiality of all participants was maintained throughout the study. Unique codes for participant identification were used, data were stored in password-protected computers and files, and data transmitted electronically were securely encrypted. Facebook uses an automatic advertising system, in which no individual user’s information is revealed to the advertiser. After clicking on an advertisement, users of Facebook were automatically directed to our secure study website, and all subsequent study procedures took place outside Facebook. This approach minimized the amount of information exchanged via the Facebook website, to further ensure the privacy and security of participants’ information.

## Results

### Recruitment

The Facebook advertisements were displayed 36,154,610 times, resulting in 8339 clicks on the advertisement (which directed respondents to the study website) and 551 expressions of interest submitted through our website. The number of times an advertisement was displayed to a unique Facebook user was 469,678, resulting in 7940 unique clicks (some Facebook users clicked on the advertisement multiple times, bringing the total number of clicks to 8339). In total 65.69% (3121/4751) of logged visits to our website lasted less than 10 seconds, 12.0% (568/4751) lasted between 10 seconds and 1 minute, and 22.35% (1062/4751) lasted for more than 1 minute. The About This Study webpage, containing details about the aims of the study and eligibility criteria, received 1144 unique visitors, who spent an average of 53 seconds on the page.

**Figure 2:**
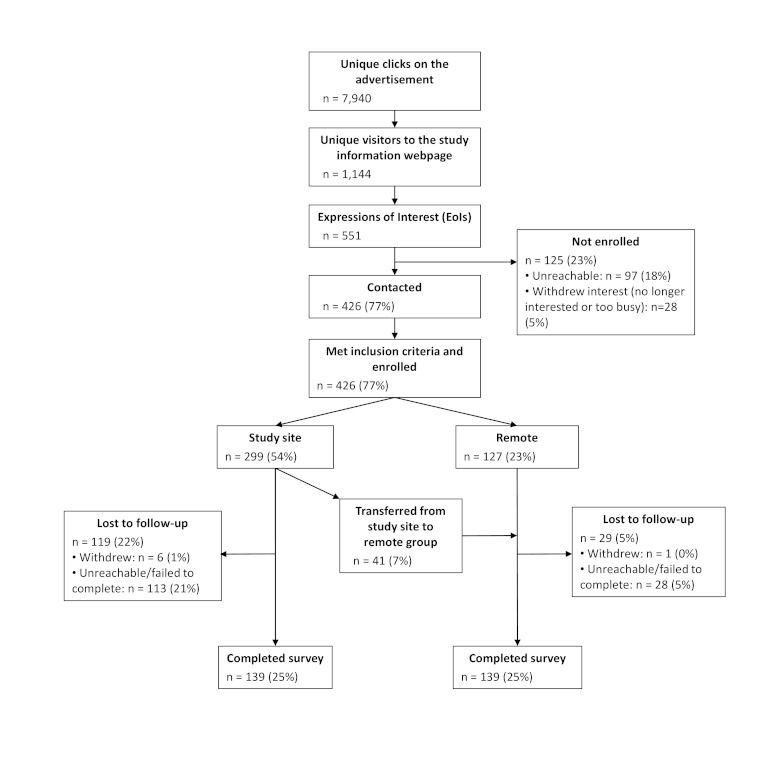
Summary of sampling and response. Percentages are calculated using the number of expressions of interest (551) as the denominator.

Of the 551 participants who initially responded to an advertisement, 426 were contactable by telephone and enrolled in the study (none was excluded due to not meeting the eligibility criteria), and 278 completed the survey (Figure 2), which took most participants 15–30 minutes to complete. Thus, the participation rate for those who clicked on the advertisement was 3.5% (278/7940), and the participation rate for those who read about the study from the About This Study webpage was 24.3% (278/1144). The average Facebook charge was US $0.67 per click, amounting to $10.16 per expression of interest, or $20.14 per compliant participant. Age and geographic region were not strong predictors of the likelihood of clicking on the advertisement. However, for those who did click on the advertisement, older age was predictive of submitting an expression of interest (Table 1). Because of this, as well as differences in average bids for each campaign, the average cost per participant varied with age group ($15, $23, and $49 per participant for 22- to 25-, 18- to 21-, and 16- to 17-year-olds, respectively, using data from June 30, 2010 onward, when there were separate advertising campaigns for the different age groups).

The age distribution of the 551 initial respondents who submitted an expression of interest reflected the general population (Table 2). This was achieved despite the lower odds of 16- to 17-year-olds submitting an expression of interest after clicking on the advertisement, by targeting the Facebook campaign budget to elicit more clicks from 16- to 17-year-olds. The broad geographic distribution, measured by Remoteness Area [29], of the 328 (59.5%) initial respondents who provided their location revealed moderate overrepresentation of regional/rural females.

**Table 1:** Prevalence ratios of clicking on the Facebook advertisement and submitting an expression of interest

Characteristic		Clicking on an advertisement			Expression of interest		
		Adjusted PR^a^	95% CI^b^	*P* value	PR^c^	95% CI	*P* value
**Age group (years)**							
	16–17	1.00			1.00		
	18–21	1.05	0.99–1.11	.10	2.42	1.85–3.19	<.001
	22–25	1.14	1.07–1.21	<.001	3.34	2.56–4.36	<.001
**Geographic region**							
	Major city	1.00					
	Regional/rural	0.96	0.90–1.04	.36			

^a^ Prevalence ratios (PRs) of clicking on a Facebook recruitment advertisement, mutually adjusted for geographic region and age group.

^b^ Confidence interval.

^c^ PRs of submitting an expression of interest, after clicking on an advertisement. Geographic region is omitted from this analysis because 40.5% (223/551) of respondents did not provide this information.

**Table 2:** Demographic characteristics of respondents who submitted an expression of interest

Characteristic		Respondents (n = 551)			Target population^b^	Fisher’s exact *P* value
		n	%	95% CI^a^		
**Age group (years)**						
	16–17	98	18%	14.6–21.0	19.8%	
	18–21	217	39.4%	35.3–43.5	40.1%	
	22–25	236	42.8%	38.7–47.0	40.1%	0.34
**Geographic region**^c^						
	Major city	238	72.6%	67.7–77.4	78.7%	
	Inner regional	72	22%	17.5–26.4	17.7%	
	Outer regional/remote	18	6%	3.0–8.0	3.6%	0.02

^a^ Confidence interval.

^b^ Population data from Australian Bureau of Statistics census 2006, with figures corrected for nonresponses to add up to 100%.

^c^ Only 328 of the 551 respondents provided geographic region information.

### Participant Characteristics

Predictors of participation were older age, higher education level, and higher BMI (calculated from self-reported height and weight), as compared with the general target population (Table 3). Females born outside Australia and those from regional and lower socioeconomic areas were well represented, with these variables not associated with likelihood of participation.

Three participants identified themselves as Aboriginal or Torres Strait Islander Australians (1.08%, 95% CI 0.06–2.10), which is consistent with the 0.85% in the target population (*P* = .68).

Initial respondents 18–25 years old were more likely to be enrolled in the study and complete the survey than younger respondents (PR = 1.37, 95% CI 1.05–1.78, *P* = .02). This is associated with a higher proportion of 16- to 17-year-olds being unreachable, even using a combination of telephone calls, SMS, and email (29% (28/98) vs 15% (69/453), *P* = .003). Of the respondents who were contactable and enrolled, the completion rate did not vary by age group (*P* = .6).

The strongest predictor of willingness to travel to the study site to complete the survey, compared with completing it online from a remote location, was proximity to the study site, as measured by geographic region (Table 4). As a result, people from major cities were overrepresented in the study site population. However, the overall study population (study site plus remote) was geographically representative. Participants from areas with postal/zip codes in the highest bracket of socioeconomic advantage were 50% more likely to visit the study site than those in the lowest bracket, although there was no such difference for participants in the middle bracket (Table 4). This may be associated with proximity to the study site. Considering only the females living in the major cities region (in which our study site was located), the mean distance to the study site from participants’ postal codes was 14, 29, and 39 km for those in the highest, middle, and lowest socioeconomic bracket, respectively. Furthermore, of the 211 participants in the major cities region, 78% (51/65; 95% CI 68%–88%) of those living within 10 km of the study site travelled to the study site to complete the survey, compared with only 51% (75/146; 95% CI 43%–59%) of those living further than 10 km from the study site. Indeed, when we included distance to the study site as a variable in our model, it was a significant predictor of attending the study site (PR = 0.92, 95% CI 0.84–0.99, *P* = .04, where each 10 km increase in distance from the study site corresponds to a factor 0.92 decrease in PR of visiting the study site, holding all other variables in the model constant), while geographic region and socioeconomic bracket were no longer significant.

Participants classified as overweight according to their BMI were less likely to travel to the study site than those with normal BMI, although participants who were obese and those with normal BMI were equally likely to visit the study site (Table 4). The prevalence of overweight/obesity (derived using the World Health Organization classifications of adult body weight status based on BMI [34]) increased with age, with 24% (9/37; 95% CI 10.5–38.1) of 16- to 17-year-olds, 33% (36/108; 95% CI 24.4–42.2) of 18- to 21-year-olds, and 36% (44/121; 95% CI 27.8–44.9) of 22- to 25-year-olds being overweight or obese. From a linear regression model, each added year in age corresponded to an increase in BMI of 0.29 kg/m^2^ (95% CI 0.06–0.52, *R* *2* = .02, *P* = .01). Those from regional/rural areas were also more likely to be overweight or obese, with prevalences of 31% (63/203; 95% CI 24.7–37.4), 40% (18/45; 95% CI 25.7–54.3), and 50% (8/16; 95% CI 25.5–74.5) for major city, inner regional, and outer regional/remote areas, respectively.

The study questionnaire also asked nonurban participants to rate the likelihood that they would have a “physical examination and/or tests once per year for 4 years as part of a health study if” (1) “we paid for your travel to and from a study site in Melbourne and accommodation for up to two nights,” and (2) “we travelled to your town to conduct the physical examination.” These items were measured on a 5-point Likert-type scale, from 1 (not at all likely) to 5 (very likely). The mean rating was 4.49 (SD 0.84) for scenario 1 and 4.50 (SD 0.82) for scenario 2, indicating that these options were equally acceptable to nonurban participants.

Less than 5% of survey data were missing on the demographic variables presented, while less than 8% of data were missing on any single variable.

**Table 3:** Demographic characteristics of participants

Characteristic		Study population (n = 278)			Target population^c^	Fisher’s exact *P* value
		n^a^	%	95% CI^b^		
**Age group (years)**						
	16–17	38	14%	9.60–17.7	19.8%	
	18–21	115	41.4%	35.5–47.2	40.1%	
	22–25	125	44.9%	39.1–50.8	40.1%	.02
**Geographic region**						
	Major city	211	76.5%	71.4–81.5	78.7%	
	Inner regional	49	18%	13.2–22.3	17.7%	
	Outer regional/remote	16	6%	3.0–8.6	3.6%	.15
**Country of birth**						
	Australia	230	83.3%	78.9–87.8	80.2%	
	Other	46	17%	12.2–21.1	19.8%	.23
**Indigenous status**						
	Aboriginal or Torres Strait Islander	3	1%	0.06–2.10	0.85%	
	Other	275	98.9%	97.9–99.9	99.15%	.51
**Socioeconomic level (SEIFA percentile)**^d^						
	<55	83	30%	24.6–35.5	33.9%	
	55–80	87	32%	26.0–37.0	32.7%	
	>80	106	38.4%	32.6–44.2	33.4%	.19
**Education level**						
	< Year 12^e^	54	19%	15.5–23.3	28.4%	
	Year 12	104	37.4%	32.6–42.2	38.1%	
	> Year 12	120	43.2%	37.3–49.0	33.5%	<.001
**Body mass index (kg/m^2^), 18- to 24-year-olds**^f^						
	<18.5 (underweight)	13	6%	3.6–8.4	9.6%	
	18.5–25 (normal)	128	61.0%	55.6–65.5	68.0%	
	25–30 (overweight)	44	21%	16.6–24.8	15.8%	
	>30 (obese)	25	12%	9.4–16.2	6.6%	.002

^a^ Numbers may not add up to 278 due to missing data.

^b^ Confidence interval.

^c^ Population data from Australian Bureau of Statistics census 2006, except for body mass index data from the Victorian Population Health Survey 2008, with figures corrected for nonresponses to add up to 100%.

^d^ Based on postal/zip code. Percentiles are the rankings within Victoria. Note that the percentiles are based on the postal codes and are not weighted by the population within each postal code. Major city postal codes have, on average, higher Socio-Economic Indexes for Areas (SEIFA) level and larger population than regional postal codes. Consequently, the population-weighted median SEIFA percentile is about 70%, not 50%.

^e^ Year 12 is the final year of high school in the Australian education system.

^f^ Age range chosen to match that from Victorian Population Health Survey 2008. Consistent with the Survey, we used the World Health Organization classifications of adult body weight status based on body mass index.

**Table 4:** Associations between completing^a^ the survey at the study site and completing it remotely, by sociodemographic characteristics

Characteristic		Completed remotely (n = 139)^b^	Completed at study site (n = 139)^b^	Adjusted PR of visiting study site^c^	95% CI^d^	*P* value
**Age group (years)**						
	16–17	26 (19%)	12 (8.6%)	1.00		
	18–21	49 (35%)	66 (47.5%)	1.32	0.8–2.19	.28
	22–25	64 (46%)	61 (43.9%)	1.15	0.68–1.95	.60
**Geographic region**						
	Major city	85 (62%)	126 (90.7%)	1.00		
	Inner regional	38 (28%)	11 (7.9%)	0.46	0.25–0.85	.01
	Outer regional/remote	14 (10%)	2 (1.4%)	0.26	0.07–0.98	.05
**Country of birth**						
	Australia	114 (83.2%)	116 (83.5%)	1.00		
	Other	23 (17%)	23 (16.5%)	0.88	0.65–1.21	.44
**Socioeconomic level (SEIFA percentile)**^e^						
	<55	59 (43%)	24 (17.3%)	1.00		
	55–80	46 (34%)	41 (29.5%)	1.09	0.73–1.63	.68
	>80	32 (23%)	74 (53.2%)	1.48	1.03–2.13	.03
**Education level**						
	< Year 12^f^	37 (27%)	17 (12.2%)	1.00		
	Year 12	41 (30%)	63 (45.3%)	1.34	0.86–2.10	.20
	> Year 12	61 (44%)	59 (42.5%)	1.15	0.72–1.84	.55
**Body mass index (kg/m^2^)**						
	<18.5 (underweight)	12 (9%)	4 (3.0%)	0.49	0.22–1.10	.08
	18.5–25 (normal)	70 (53%)	91 (67.9%)	1.00		
	25–30 (overweight)	33 (25%)	22 (16.4%)	0.72	0.53–0.98	.04
	>30 (obese)	17 (13%)	17 (12.7%)	1.05	0.74–1.50	.78

^a^ For the purposes of this study, we define a survey as being complete if 80% of the demographic information needed for our analysis was provided. A total of 5 participants did not fully complete the survey, but did provide most of the demographic data used in our analyses.

^b^ Numbers may not add up to 278 due to missing data.

^c^ Prevalence ratios (PRs) of visiting study site to complete the survey, versus completing online remotely. Poisson regression models were mutually adjusted for age group, geographic region, country of birth, socioeconomic level (Socio-Economic Indexes for Areas [SIEFA] percentile), education level, and body mass index. Small numbers of indigenous females in our sample did not support meaningful analyses and thus indigenous status was excluded from this model.

^d^ Confidence interval.

^e^ Socio-Economic Indexes for Areas (SEIFA) percentile, based on postal/zip code.

^f^ Year 12 is the final year of high school in the Australian education system.

## Discussion

This study demonstrated good levels of engagement of young females, who are traditionally underrepresented in health studies or have poorer access to health care. For example, the strong representation of regional and rural females in this study shows the potential benefit of using social networking sites to recruit a segment that traditionally has been quite difficult to reach. Rural and regional participants were less likely to travel to the study site, which in most cases would have involved round trips of 2 to 8 hours. Nonetheless, a representative study site population could be obtained by oversampling nonurban females. Moreover, the survey results indicate that study site compliance rates may be increased by providing sites close to participants’ place of residence or offering accommodation and compensation for regional participants to travel to an urban study site.

We obtained a representative distribution of Australian and non-Australian-born participants, which compares favorably with many population-based studies where overseas-born participants are underrepresented. For instance, in the Australian Longitudinal Study on Women’s Health (ALSWH), which used mail-out invitations in 1996 to recruit women randomly selected from the Medicare database, 88.6% of 18- to 23-year-old respondents were Australian-born versus 77.8% in the target population [35]. More recently, in the Victorian Population Health Survey 2008, which used random digit dialing to sample from residential households with landline telephone connections, 79.2% of respondents were Australian-born versus the target of 71.3% [30]. On the other hand, more highly educated females were overrepresented in our sample, which is another common bias in population-based studies (eg, the ALSWH, where 18- to 23-year-old respondents were more likely than the target population to be tertiary educated) [35]. The overrepresentation of more highly educated females in our study population may be overstated in the data. It is likely that some participants misunderstood the question about education level (“What is your highest level of education completed?”) and indicated the level of education they are currently *completing*, rather than their highest level *completed*. For instance, of the 33 participants who indicated that they were currently attending high school, 8 (24%) answered that their highest level of education completed was Year 12. However, if that were the case, they would no longer be attending high school. We corrected these data, but we could not identify inconsistent answers for other educational levels, since someone may have, for example, completed a university degree and still be attending university.

Respondents 16–17 years old were less likely than those 18–25 years old to be enrolled in the study. The lower participation rate of 16- to 17-year-olds was associated with their being harder to contact, even using a combination of calling their mobile/cellular telephone and sending SMS messages and emails. A contributing factor may have been that the delay between the expression of interest and first attempt at contacting the 16- to 17-year-olds was, on average, 3 weeks longer than for 18- to 25-year-olds. This delay was due to the logistics of performing the mature minor assessment.

Overweight and obese young females were strongly represented. The average BMI (based on self-reported weight) in our study population was higher than that of the target population, with 33% (69/210) of 18- to 24-year-olds being overweight or obese, compared with 22% in the target population (*P* < .001). Previous reports suggest that Internet and interactive media use is positively correlated with BMI in adults [36] and adolescent females [37]. Our findings demonstrate the potential utility of Facebook as a recruitment tool in these high-risk young females, who may find initial online engagement less confrontational than other approaches. Notably, the prevalence of overweight and obesity combined rose appreciably across age groups, which is consistent with major lifestyle and health changes occurring at this stage of life. This observation also lends support to the urgent need for more research into evolving health risks in young women.

Social networking site recruitment shows great potential to yield a demographically representative sample by oversampling and appropriately weighting data. The ability to create multiple advertising campaigns targeted to different populations, and to closely monitor their real-time performance, allows one to reallocate resources between campaigns to direct recruitment efforts toward targeted parameters such as age and location of residence. Moreover, it seems clear from our findings that this approach could be used to direct recruitment campaigns to people with particular health problems or health risks.

At an average of US $20 in advertising fees per participant, this recruitment method compares favorably with traditional methods, which can cost US $20–$500 per participant, depending on the particular strategy and target population [3,4,38,39]. Traditional passive recruitment through paid media campaigns (eg, radio, television, and newspaper advertising) may have broad reach throughout the community but can be expensive [3] and ineffective, as proportional use of these media has decreased over the years compared with Internet use. Active face-to-face recruitment (eg, through schools, community groups, and health professionals) is generally labor intensive, expensive, and unsuitable for obtaining a representative population. Random digit dialing and direct mail that draws on information from electoral lists or health care databases have been widely used to obtain population-based samples, but as with other active recruitment methods, they are more labor intensive and costly than passive Facebook recruiting [40]. Random digit dialing telephone sampling has been a very popular recruitment and survey tool; however, its coverage is decreasing as fewer households have active landline telephones. In 2008, 16% of all US adults, 63% of adults in shared households (living with nonrelatives), and 31% of 18- to 24-year-olds lived in mobile/cellular phone-only households [2]. In Australia in 2010, 33% of all 18- to 24-year-olds had no landline telephone in their household, and for those living outside the parental home, the figure was almost 60% [41]. This trend is occurring worldwide, threatening the generalizability of studies that employ random digit dialing of landlines only. There have been promising results from random digit dialing studies that include mobile/cellular phones, but the costs were up to 5 times higher than for landline random digit dialing [42]. It should be noted that Facebook advertising costs are likely to increase, particularly as it grows in popularity, with more advertisers competing for ad space and driving up the bid price. Indeed, the cost-per-click rates in the United States, United Kingdom, France, and Germany rose 74% in the 12 months from the second quarter of 2010 to the second quarter 2011 [43].

### Strengths and Limitations of the Study

A limitation of this recruitment method is the low participation rate and the potential for volunteer bias. The participation rate of those who visited our About This Study webpage and had an opportunity to read about the study and make an informed decision about joining was 24%, while the participation rate of those who clicked on the Facebook advertisement was only 3.5%. This is lower than typical rates for population-based studies using traditional recruitment methods. For instance, using mail-out recruitment, the ALSWH had a response rate of about 40% in its youngest cohort [35], which is typical for mail-out survey response rates [44]. ALSWH compared their sample demographics with census data to confirm that the participants were reasonably representative of the general population. Despite their higher response rate, they actually found evidence for a bigger response bias than in our study, and in particular, a more serious overrepresentation of tertiary-educated females (12.1% had completed a university degree vs 7.7% in the target population) and Australian-born females (88.6% vs 77.8%). Broad demographics aside, there may be biases in our sample that we did not measure with our survey—for instance, psychological, social, or familial factors—although we did use the SEIFA as an approximation of socioeconomic advantage and found no difference between the study and target populations. Further studies are needed to investigate other predictors of participation, and information on reasons for nonparticipation would also be informative.

Other limitations include bias due to exposure to the advertisement being positively correlated with time spent on Facebook (and therefore not the same for each user), the need for users to supply their correct gender and age in their profile in order to be exposed to the advertisement, and chain sampling bias, whereby users exposed to the advertisement may share information about the study with others who may then submit an expression of interest. We were able to evaluate the latter phenomenon and found that it did not meaningfully change our results. Specifically, 25 participants indicated that they found out about the study through a friend or relative. Exclusion of these females from the analyses did not result in any significant change in the results. We did not omit them from the main analysis because in observational studies, particularly in this age group and in this era of rapid information sharing, we expect this to be a common occurrence, although it is not always measured in research studies.

This study was conducted in the state of Victoria, Australia, and further research is needed to determine how applicable our findings are to health research internationally. Several factors suggest that our results may be more broadly generalizable. In particular, most other developed nations are also witnessing the phenomenon of very high rates of social networking site usage along with declining landline telephone prevalence, which together make this a very promising recruitment approach that is readily portable to other regions and countries. Moreover, as above, social networking site use in developing countries is expanding at very high rates, suggesting the potential to use this approach to recruitment for biomedical research in many parts of the world.

As this was a cross-sectional study, we were unable to measure retention rates, which would have informed applications of this sampling frame to longitudinal studies. There was a high level of self-reported willingness to answer questionnaires and have physical examinations, tests, and sample collections in an ongoing health study. Indeed, nearly all respondents stated that they would like to be contacted about participating in a large longitudinal study about young women’s health.

Despite the above limitations, the study population was demographically similar to the general population of 16- to 25-year-old Victorian females. We obtained a large sample size (n = 278) for the recruitment period and achieved high compliance in completing the survey with very little missing data, and the method was highly cost effective. In addition, this method allows researchers to very precisely extract the components of responses (clicks on the advertisement, navigation around the website, expression of interest, etc), whereas many other recruitment methods do not.

### Conclusions

Results from this study suggest that targeted recruitment using the social networking site Facebook has strong potential for yielding demographically representative samples of young females, a population far more likely to engage with the health community using Facebook than landline telephones. A substantial majority of participants expressed clear willingness to participate in extensive longitudinal studies of their health. This model may also be appropriate for recruiting other populations, as use of social networking sites by older people and minorities continues to grow [45,46], as well as for intervention studies and clinical trials. Furthermore, this strategy was cost effective in comparison with many traditional methods.

## Multimedia Appendix 1

Study questionnaire.

## Abbreviations

ALSWH: Australian Longitudinal Study on Women’s Health
BMI: body mass index
CI: confidence interval
PR: prevalence ratio
SEIFA: Socio-Economic Indexes For Areas
