# Supplementary material for: Web-Based Recruiting for Health Research Using a Social Networking Site: An Exploratory Study
Source: J Med Internet Res. 2012 Feb 1;14(1):e20. doi: 10.2196/jmir.1978 (PMC3374531; doi:10.2196/jmir.1978)
Supplement: Supplementary file 1 [file jmir_v14i1e20_app1.pdf]

YFHI questionnaire

Information and Consent

Please read the information provided below carefully. If you have any questions or concerns at this point or in the future, please contact any of the people listed below.

WE ENCOURAGE YOU TO CONTACT US AT ANY TIME WITH QUESTIONS, FEEDBACK, CONCERNS OR COMPLAINTS.

Name: YFHI study coordinators
Telephone: 1800 188 385

Name: Professor Suzanne Garland
Role: Director, Royal Women's Hospital Department of Microbiology and Infectious Diseases
Telephone: (03) 8345 3671

Name: Dr. Yeshe Fenner
Role: Project Officer, Royal Women's Hospital Department of Microbiology and Infectious Diseases
Telephone: (03) 8345 3682

Name: Dr. Elya Moore
Role: Senior Clinical Epidemiologist, Royal Women's Hospital Department of Microbiology and Infectious Diseases
Telephone: (03) 8345 3692

For complaints:
Name: Consumer Advocate
Telephone: (03) 8345 2290

WHAT IS THE PURPOSE OF THIS STUDY?

The aim of this research is to see if researchers can use social networking sites like Facebook to recruit young Victorian women into a health and lifestyle study. We also want to find out about young women's health, behaviour and medical history, and their attitudes towards health research and a range of health issues.

WHAT WILL I NEED TO DO AS A PARTICIPANT?

You will be asked to complete a questionnaire that asks about your health, behaviour and medical history, and your attitude towards health research and a range of health issues. This questionnaire will take bout 15-20 minutes to complete.

WHAT ARE THE RISKS AND BENEFITS TO ME?

The risks associated with participation in this study are minimal. A small risk to you is that some of the questions may make you feel embarrassed or upset. If this happens, please contact us using the contact information above, to provide feedback to us. We can also arrange appropriate support or counselling.

There may not be a direct benefit to you personally for participating in this study. However, results from this study will help us understand how to recruit young women into research. By participating in this study you will be contributing to the design and focus of future studies in young women.

ARE MY ANSWERS CONFIDENTIAL?

Any information obtained in connection with this research project that can identify you will remain confidential and will only be used for the purpose of this research project. The only people with access to your information are the researchers for the study.

WHAT IF I DON'T WANT TO PARTICIPATE?

YFHI questionnaire

Your participation in this study is completely voluntary and you can withdraw or discontinue the survey at any time.

PARTICIPANT'S STATEMENT OF INFORMED CONSENT:

If you agree with the following statement and wish to participate in the study, please click on the circle in front of "I agree" below.

If you do not agree, simply close your browser.

1. "I am female, I am 16-25 years of age, I live in Victoria (Australia), I understand the explanation provided to me and voluntarily agree to participate in this study."

◯ I agree

PRINT THIS PAGE FOR FUTURE REFERENCE.

SECTION A: Please tell us about yourself

2. Please enter the participant ID you received from the study coordinators.

3. What is your date of birth?

DD MM YYYY

(Day/Month/Year) / /

4. Are you single or in a relationship? (Mark one answer)

- Single
- Couple not living together
- Couple living together
- Prefer not to answer
- Other (please specify)

5. What is your present marital status?

- Never married
- Widowed
- Divorced
- Separated but not divorced
- Married
- Prefer not to answer
- Other (please specify)

6. It is common for living arrangements to vary these days. Which of the following best describes your household structure? (Mark one answer)

- ◯ Living alone
- ◯ Living with flat or housemates
- ◯ Living with parents
- ◯ Living as a couple without children
- ◯ Living as a couple living with children
- ◯ Single parent/guardian living with children
- ◯ Prefer not to answer

Other (please specify)

7. What is your highest level of education completed? (Mark one answer)

- ◯ Did not go to school
- ◯ Year 8 or below
- ◯ Year 9 or equivalent
- ◯ Year 10 or equivalent
- ◯ Year 11 or equivalent
- ◯ Year 12 or equivalent
- ◯ Certificate, diploma or advanced diploma, e.g. Technical or further education institution (TAFE)
- ◯ Undergraduate degree (e.g. Bachelor)
- ◯ Graduate diploma or Graduate certificate
- ◯ Postgraduate degree
- ◯ Prefer not to answer
- ◯ Other (please specify)

8. Are you currently attending school or any other educational institution?

- ◯ No
- ◯ Yes, full-time student
- ◯ Yes, part-time student

SECTION A) Educational institution

9. What type of educational institution are you attending? (Mark one answer)

- ◯ High School / Secondary School
- ◯ Tertiary Institution (e.g. University or TAFE)
- ◯ Other (please specify)

SECTION A) High school

10. What type of High School / Secondary School are you attending?

- ◯ Public/Government
- ◯ Catholic non-government
- ◯ Other non-government (private)

SECTION A) Tertiary institution

11. What type of tertiary institution are you attending?

- ◯ Technical or further education institution (e.g. TAFE college)
- ◯ University or other higher education institution
- ◯ Other (please specify)

SECTION A) Employment

12. How much money do you earn in an average week before tax? (Mark one answer)

- ◯ No income or negative income
- ◯ $1-$149
- ◯ $150-$249
- ◯ $250-$399
- ◯ $400-$599
- ◯ $600-$799
- ◯ $800-$999
- ◯ $1,000-$1,299
- ◯ $1,300-$1,599
- ◯ $1,600-$1,999
- ◯ $2,000 or more
- ◯ Prefer not to answer

13. What is your job status? (Mark one answer)

- ◯ Employed full-time
- ◯ Employed part-time
- ◯ Employed but away from work (e.g. on holidays or paid leave)
- ◯ Unemployed and looking for work
- ◯ Not in the labour force (not looking for work because e.g full-time student, mother)
- ◯ Prefer not to answer

SECTION A) location and ancestary

14. What is the postal code of your usual residence?

15. Where have you lived for most of your life?

Suburb/city:

State:

Postcode:

Country:

Additional comments:

16. During your whole life, how many years in total have you lived on a farm?

- 0 years
- 1-5 years
- 5+ years

17. What country was your mother born in? (Mark one answer)

- Australia
- United Kingdom
- New Zealand
- Italy
- China
- Prefer not to answer
- Other (please specify)

18. What country was your father born in? (Mark one answer)

- Australia
- United Kingdom
- New Zealand
- Italy
- China
- Prefer not to answer
- Other (please specify)

19. What country were you born in? (Mark one answer)

- ◯ Australia
- ◯ England
- ◯ New Zealand
- ◯ Italy
- ◯ China
- ◯ Prefer not to answer
- ◯ Other (please specify)

20. Do you speak a language other than English at home? (Mark one answer)

- ◯ No, English only
- ◯ Yes, Italian
- ◯ Yes, Greek
- ◯ Yes, Cantonese
- ◯ Yes, Arabic
- ◯ Yes, Vietnamese
- ◯ Yes, Mandarin
- ◯ Prefer not to answer
- ◯ Other (please specify)

21. What is your ancestry? (Consider the origins of your parents and grandparents) (Mark up to two answers)

- ☐ English
- ☐ Irish
- ☐ Italian
- ☐ Greek
- ☐ German
- ☐ Chinese
- ☐ Scottish
- ☐ Australian
- ☐ Prefer not to answer
- ☐ Other (please specify)

22. Are you of Aboriginal or Torres Strait Islander origin? (Mark up to two answers)

- ☐ No
- ☐ Yes, Aboriginal
- ☐ Yes, Torres Strait Islander
- ☐ Yes, Aboriginal and Torres Strait Islander
- ☐ Prefer not to answer

SECTION A) continued

23. How did you hear about our study? (Mark all that apply)

- ☐ An advertisement on Facebook
- ☐ An advertisement on MySpace
- ☐ An advertisement on Google
- ☐ A group page on Facebook
- ☐ A group page on MySpace
- ☐ A friend shared the group page with you on Facebook
- ☐ A friend shared the group page with you on MySpace
- ☐ Other (please specify)

24. What sort of technology do you use to socialize online (e.g. Facebook, MySpace)? (Mark all that apply)

- ☐ Mobile phone
- ☐ Personal computer
- ☐ Shared computer at home
- ☐ Work computer
- ☐ Internet cafe
- ☐ Other (please specify)

25. Do you have any of the following forms of identification? (Mark all that apply)

- ☐ Driver's license
- ☐ Student ID card
- ☐ Medicare card
- ☐ Other (please specify)

SECTION A) continued

26. How tall are you without shoes? (If you are not sure, please estimate)

If you prefer not to answer, you can skip to the next question

in cms

or in feet and inches

27. How much do you weigh without clothes or shoes? (If you are not sure, please estimate)

If you prefer not to answer, you can skip to the next question

in kgs

28. Are you currently pregnant?

- Yes
- No
- Don't know
- Prefer not to answer

29. Are you currently breastfeeding?

- Yes
- No
- Prefer not to answer

SECTION B)

We would like to ask you questions about how you feel about answering private questions about yourself. We will also ask whether you would feel comfortable with certain types of research procedures.

30. Please fill in the circle that best represents your comfort level on a scale of 1 to 5 about answering certain questions. You may or may not have had any experiences with the following issues. We would just like to know how comfortable you would be if we asked you more detailed questions about these issues.

Comfort level answering a question about:

	1 = not comfortable at all	2	3	4	5 = completely comfortable
Medical history (this means that we would ask you about whether or not you have any medical conditions or are taking medicines, or have had surgery in the past)	○	○	○	○	○
Age of first sexual intercourse	○	○	○	○	○
Lifetime number of sexual partners	○	○	○	○	○
Condom use	○	○	○	○	○
Oral sex	○	○	○	○	○
History of sexually transmitted infections	○	○	○	○	○
Having sex against your will	○	○	○	○	○
Violence in the home	○	○	○	○	○
Unwanted sexual experiences in childhood	○	○	○	○	○
Mental health (for example depression and anxiety)	○	○	○	○	○
Age of first menstrual period	○	○	○	○	○
Period problems (i.e. irregular bleeding, heavy bleeding, period pain)	○	○	○	○	○
Birth control pills (same as oral contraception pills)	○	○	○	○	○
Details regarding pregnancies	○	○	○	○	○
Details regarding miscarriages	○	○	○	○	○
Details regarding pregnancy terminations (abortions)	○	○	○	○	○
Alcohol use	○	○	○	○	○
Smoking	○	○	○	○	○
Use of alternative medicine (e.g. herbal medicine)	○	○	○	○	○
Use of recreational drugs (e.g. marijuana, “party” drugs)	○	○	○	○	○
Use of vitamin supplements (e.g. vitamin D, vitamin C)	○	○	○	○	○
Use of over the counter medications (e.g. panadol)	○	○	○	○	○
Use of prescription medications	○	○	○	○	○
The amount of time spent doing physical activity	○	○	○	○	○

31. How you would prefer to complete an online questionnaire that takes 2 hours to finish, and would be given annually for 4 years? (Mark one answer)

- ◯ All at once
- ◯ Spread over 2 days
- ◯ Spread over 5 days
- ◯ Spread over 10 days
- ◯ Spread over 15 days

Other (please specify)

32. Please fill in the circle that best represents your comfort level on a scale of 1 to 5 about going through certain medical tests with a study nurse (Note that you will not have these procedures done as part of this study).

Comfort level undergoing a:

	1 = not comfortable at all	2	3	4	5 = completely comfortable
Physical examination where we listen to your heart and check your weight, height, blood pressure, heart rate, and waist size.	○	○	○	○	○
A blood test so we can measure fat, sugar, hormones, and immune cells in your blood.	○	○	○	○	○
A urine test to see if you are pregnant.	○	○	○	○	○
A fitness test to see how fit you are. This test would involve things such as walking, stretching, and push ups.	○	○	○	○	○
A bone density scan to check how strong your bones are, and what percentage of your body is made up of fat. This would be done twice, with two different machines. For the first one, you would be asked to lie down on a table and hold very still for a few minutes. For the second one, which also takes a few minutes, you would be seated and the scanner would measure bone strength in your tibia (a leg bone) Each test involves exposure to a very small amount of X-rays.	○	○	○	○	○
Would you be comfortable repeating the bone density scan tests in another 2 years?	○	○	○	○	○
Would you feel more comfortable if you discussed the bone density scan test with your parents beforehand?	○	○	○	○	○
A sexually transmitted infections test that includes looking for chlamydia, human papillomavirus (HPV) on a vaginal swab (such as a cotton bud) and oral swab. Chlamydia is a very common sexually transmitted infection affecting young women, which can be treated with antibiotics. Most people with chlamydia do not know that they have it. Both chlamydia and HPV can be tested for using a vaginal swab that you can learn to take yourself, without having to undergo a genital examination by a nurse or doctor.	○	○	○	○	○
Would you feel more comfortable if you discussed the sexually transmitted infections test with your parents beforehand?	○	○	○	○	○
A genital examination comprised of a Pap (collecting cells from your cervix) smear, checking your genital area for warts, and a pelvic examination	○	○	○	○	○
Would you feel more comfortable if you discussed the genital examination with your parents beforehand?	○	○	○	○	○

SECTION B) continued

33. Please fill in the circle that best represents your willingness to do certain study procedures on your own.

Willingness to:

	1 = not willing at all	2	3	4	5 = completely willing
Come to a two-hour appointment at a study clinic in Melbourne once a year	◯	◯	◯	◯	◯
Collect a vaginal swab (such as a cotton bud) from your vagina: a swab is similar to a bud/cotton tip. We would provide instructions on how to insert this into the lower vagina, then place into a container for testing.	◯	◯	◯	◯	◯
Collect an oral swab from your mouth	◯	◯	◯	◯	◯
Keep a detailed food diary for one week	◯	◯	◯	◯	◯
Take digital pictures of your meals before and after you eat them and send the pictures to us. We would ask you to do this for all of the food that you eat in a given day, for a total of 4 days.	◯	◯	◯	◯	◯
Answer a one hour questionnaire and mail it in a pre-paid envelope	◯	◯	◯	◯	◯
Answer questions and surveys by text	◯	◯	◯	◯	◯
Answer questions and survey by the internet on a secure server	◯	◯	◯	◯	◯
Join a Facebook or MySpace group page with other young women in the study	◯	◯	◯	◯	◯
Use the Nintendo “Wii” to measure fitness at your home. Some of the components of fitness that can be measured on the Wii are: the amount of time spent on the Wii, the activities played, and changes in BMI (body mass index, an indicator of obesity) and posture overtime.	◯	◯	◯	◯	◯
Ask your parents for permission to participate in the study	◯	◯	◯	◯	◯

SECTION B) nutrition

Here are some typical questions that we might ask when we are assessing your nutrition.

34. Do you drink milk? (Mark only one)

- ◯ Yes
- ◯ No

SECTION B) nutrition continued

35. What brand of milk do you drink? (Mark all that apply)

- ☐ Pura
- ☐ Paul's
- ☐ Pure Organic
- ☐ Physical
- ☐ Dairy Farmers
- ☐ Rev
- ☐ Anlene
- ☐ So Good
- ☐ Vitasoy
- ☐ I don't know
- ☐ Other (please specify)

36. How often do you drink milk? (Mark only one)

- ○ Every day
- ○ Three to five times per week
- ○ One to two times per week
- ○ A couple of times a month
- ○ Hardly at all

SECTION C)

We would like to know what you are interested in gaining from a research study in young women.

37. Please fill in the circle that best represents how interested you are in each health topic.

I am interested in learning about:

	1 = not interested at all	2	3	4	5 = very interested
Sexual health (includes sexually transmitted infections)	○	○	○	○	○
Reproductive health (includes periods, irregular bleeding, and pregnancy)	○	○	○	○	○
Bone and joint health (includes how hormones, nutrition and physical activity affect your bones and risk of osteoporosis)	○	○	○	○	○
Nutrition & fitness	○	○	○	○	○
Mental health (includes depression, anxiety, drug/alcohol abuse, eating disorders)	○	○	○	○	○
Obesity	○	○	○	○	○
Diabetes	○	○	○	○	○
Heart disease	○	○	○	○	○

SECTION C) continued

38. Please fill in the circle that best represents how likely it is that you would participate in a four-year health research study if we provided you with:

	1 = not likely at all	2	3	4	5 = very likely
Results from your health and fitness tests	○	○	○	○	○
Information about sexually transmitted infections	○	○	○	○	○
Free access each year to well-trained nurses in Melbourne for health advice	○	○	○	○	○
Free access to online exercise videos	○	○	○	○	○
Free vaccine doses for the cervical cancer vaccines	○	○	○	○	○
Money to compensate for your time and travel	○	○	○	○	○
A gift certificate to a food store (such as Coles)	○	○	○	○	○
A gift certificate to the Body Shop	○	○	○	○	○
A gift certificate to a department store (such as Myers)	○	○	○	○	○
A gift certificate to the iTunes store	○	○	○	○	○
Membership to a gym	○	○	○	○	○
A Nintendo “Wii Fit”	○	○	○	○	○
An iPhone	○	○	○	○	○

39. From the items listed in the previous question, please indicate your top three choices that would make you most likely to participate in a four-year health research study.

	1st choice	2nd choice	3rd choice
Results from your health and fitness tests	○	○	○
Information about sexually transmitted infections	○	○	○
Free access each year to well-trained nurses in Melbourne for health advice	○	○	○
Free access to online exercise videos	○	○	○
Free vaccine doses for the cervical cancer vaccines	○	○	○
Money to compensate for your time and travel	○	○	○
A gift certificate to:	○	○	○
A gift certificate to a food store (such as Coles)	○	○	○
A gift certificate to the Body Shop	○	○	○
A gift certificate to a department store (such as Myers)	○	○	○
A gift certificate to the iTunes store	○	○	○
Membership to a gym	○	○	○
A Nintendo “Wii Fit”	○	○	○
An iPhone	○	○	○

SECTION C) continued

40. Do you live in Melbourne?

- ◯ Yes
- ◯ No

SECTION C) traveling to study site

41. How likely is that you would have a physical examination and/or tests once per year for four years as part of a health study if:

	1 = not likely at all	2	3	4	5 = very likely
We paid for your travel to and from a study site in Melbourne and accommodation for up to 2 nights (once per year for four years)	◯	◯	◯	◯	◯
We travelled to your town to conduct the physical examination (once per year for four years)	◯	◯	◯	◯	◯

SECTION D) The next questions are about your first sexual experiences

42. Have you had vaginal intercourse?

- ◯ Yes
- ◯ No
- ◯ Prefer not to answer

SECTION D) continued

43. How old were you when you first had vaginal intercourse?

◯ Prefer not to answer

Age (in years):

44. How old was your first partner at that time?

◯ Prefer not to answer

Age (in years):

45. What contraception precautions did you or your partner use that first time, if any?

◯ No contraception
◯ Condom
◯ Other contraception
◯ Can't remember
◯ Prefer not to answer

46. How many male sexual partners have you had in the past year (12 months)?

◯ Prefer not to answer

Number of male partners in the past year:

47. How many female sexual partners have you had in the past year (12 months)?

◯ Prefer not to answer

Number of female partners in the past year:

SECTION E) The next questions are about chlamydia and chlamydia screening

48. Have you ever heard of Chlamydia trachomatis?

- ◯ Yes
- ◯ No

SECTION E) continued

49. Have you ever had the sexually transmitted infection chlamydia?

- ◯ Yes
- ◯ No
- ◯ Prefer not to answer

50. Have you ever had a screening test for the sexually transmitted infection chlamydia?

- ◯ Yes
- ◯ No
- ◯ Prefer not to answer

SECTION E) continued

51. Why haven't you been screened for chlamydia? (Mark all that apply)

- ☐ you do not know what chlamydia is.
- ☐ you do not know what chlamydia screening is.
- ☐ you are not sexually active.
- ☐ you do not think that you need the screening test.
- ☐ you have not been offered the screening test.
- ☐ you do not have access to the screening test.
- ☐ it is too inconvenient.
- ☐ you would be too embarrassed

SECTION E) continued

52. Please answer the following questions about Chlamydia to the best of your knowledge:

	True	False	Don't Know
Chlamydia is a rare infection	◯	◯	◯
Infection with chlamydia may result in infertility	◯	◯	◯
Infection with chlamydia may result in long term pelvic pain	◯	◯	◯
Many women who are infected with chlamydia do not experience symptoms	◯	◯	◯
Chlamydia is not a serious infection	◯	◯	◯
An infection with chlamydia may be readily treated with a course of antibiotics	◯	◯	◯
Women who have had multiple sexual partners are at a higher risk of acquiring chlamydia	◯	◯	◯
Once you are treated for chlamydia, you cannot have a repeated infection	◯	◯	◯

53. Please answer the following:

	1 = completely disagree	2	3	4	5 = completely agree
I think it would be a good idea for all sexually active young women to undergo chlamydia screening annually	○	○	○	○	○
I would like to know more about chlamydia and other sexually transmitted infections	○	○	○	○	○
I would be more likely to do a chlamydia screening test if it were free	○	○	○	○	○
I would be more likely to agree to a chlamydia screening test if it were offered at the same time as a pap smear	○	○	○	○	○
I would be likely to do a chlamydia screening test if I received reminders from my GP for routine screening	○	○	○	○	○

54. Please indicate your level of comfort with the following screening methods for chlamydia:

	1 = very uncomfortable	2	3	4	5 = very comfortable
Urine sample collected when visiting the GP	○	○	○	○	○
Urine sample collected at home and then delivered to a pathology clinic or a GP	○	○	○	○	○
Self collected vaginal swab taken at the GP	○	○	○	○	○
Cervical (neck of the womb) sample taken by a GP at the GP clinic	○	○	○	○	○
Cervical sample taken at the same time as a pap smear	○	○	○	○	○

SECTION E) continued

55. Please indicate your level of comfort in attending a consultation in regards to sexual health and sexually transmitted infection screening at the following locations:

	1 = very uncomfortable	2	3	4	5 = very comfortable
Your usual GP clinic	○	○	○	○	○
A different GP clinic to the one you normally attend	○	○	○	○	○
A women's health clinic located at a hospital	○	○	○	○	○
A specialist sexual health clinic	○	○	○	○	○
Other (Please specify)	○	○	○	○	○

Other (please specify)

SECTION E) chlamydia screening

56. Would you sign up online for a chlamydia screening test? (This might entail signing up on a secure online website, receiving a cotton swab testing kit in the post, inserting the swab into your vagina, and mailing the swab to the laboratory in a prepaid envelope at no cost to you and without having to see a doctor)

- ◯ Yes
- ◯ No. If "No", why not

SECTION F) We would like to find out how much you know about vaccines for y...

57. Are you aware of any vaccines for cancer in women?

- ◯ Yes
- ◯ No

SECTION F) continued

58. Which women's cancer(s) is/are the vaccine(s) for? You can give as many answers as you'd like.

59. If you know them, please write in the name(s) of the vaccine(s) for women's cancer below. If you don't know, leave blank.

SECTION F) continued

60. Is there anything that women can do to reduce their risk of developing cervical cancer? (Cancer of the neck of the womb) Please mark all that apply.

- ☐ Have regular Pap smears
- ☐ Practice safe sex
- ☐ Get vaccinated for cervical cancer
- ☐ Not smoke
- ☐ Exercise
- ☐ Eat a healthy diet
- ☐ Nothing
- ☐ Other (please specify)

61. Have you ever heard of the vaccine for human papillomavirus (HPV)?

- ○ Yes
- ○ No

SECTION F) continued

62. Please answer the following questions about the HPV vaccine. If a woman has received the HPV vaccine, this means:

	True	False	Don't know
she does not need to have any Pap smears in the future.	◯	◯	◯
the vaccine will protect against around 80% of cervical cancers, but not the remaining 20% of cervical cancers.	◯	◯	◯
she will never have a Pap abnormality after vaccination.	◯	◯	◯
Pap abnormalities may still occur which require monitoring and treatment.	◯	◯	◯
if a Pap abnormality occurs then the vaccine has failed to do its job	◯	◯	◯

63. Where did you hear about the HPV vaccine? Please mark all that apply.

- ☐ Newspapers
- ☐ Magazines
- ☐ Television
- ☐ Radio
- ☐ Advertisement
- ☐ Government health website
- ☐ School
- ☐ Internet
- ☐ General practitioner or personal physician
- ☐ Other healthcare professional
- ☐ Friends or family
- ☐ Don't know
- ☐ Other (please specify)

____

64. Have you discussed getting the HPV vaccine with your health care provider?

- ◯ Yes
- ◯ No
- ◯ Don't remember

SECTION F) HPV vaccine

65. What about the HPV vaccine did you discuss with a health care professional? (Mark all that apply)

- [ ] Cost
- [ ] Risks
- [ ] Benefits
- [ ] Effectiveness
- [ ] Don’t remember
- [ ] Other (please specify)

SECTION F) HPV vaccine

66. Who raised the topic of an HPV vaccination? Was it...

- ◯ You
- ◯ Your health care professional
- ◯ Your school
- ◯ Other (please specify)

SECTION F) HPV

67. Do you know what HPV is?

◯ Yes

◯ No

SECTION F) knowledge about HPV

68. Please indicate if you think the following statements about HPV are true, false or you don't know.

	True	False	Don't know
It is a sexually transmitted infection	◯	◯	◯
It is a common virus that most women catch at some time in their lives	◯	◯	◯
It is an inherited (i.e. passed down from your mother or father through your genes) virus	◯	◯	◯
It is a rare virus that affects people who have had a large number of sexual partners	◯	◯	◯
It is a virus that only older people catch	◯	◯	◯
There is a link between HPV and cancer	◯	◯	◯

SECTION F) HPV vaccine history

69. Have you had the HPV vaccination (at least one dose)?

- ◯ Yes
- ◯ No
- ◯ Don't know

SECTION F) HPV doses

70. How many doses have you had?

- ◯ 1
- ◯ 2
- ◯ 3
- ◯ Not sure

71. Please fill in the circle that best indicates whether each statement is true or false.

You got an HPV vaccination:

	True	False	Don't know
to protect yourself from HPV infection and cervical cancer.	◯	◯	◯
because it was free.	◯	◯	◯
because it was offered at school.	◯	◯	◯
to join in the fight against cervical cancer.	◯	◯	◯
because your general practitioner told you it was a good idea.	◯	◯	◯

Other (please specify)

____

72. Where did you get vaccinated?

- ◯ School
- ◯ GP’s office
- ◯ Both of the above
- ◯ None of the above

SECTION F) did not get HPV vaccination

73. You did not get an HPV vaccination because...

	True	False	Don't know
you believe the vaccine is not effective.	◯	◯	◯
you believe the vaccine gives you HPV.	◯	◯	◯
you are over 26 years old.	◯	◯	◯
you have regular Pap smears.	◯	◯	◯
you are not sexually active.	◯	◯	◯
you are in a monogamous relationship (i.e. you have only one sexual partner)	◯	◯	◯
it is too inconvenient to get to the general practitioner's office for three doses.	◯	◯	◯
it costs too much.	◯	◯	◯
you are not sure that you need the vaccine.	◯	◯	◯
you are concerned about having a bad reaction to the vaccine.	◯	◯	◯
you don't like needles.	◯	◯	◯
HPV is not a big deal.	◯	◯	◯
you do not believe in getting a vaccination for a sexually transmitted infection.	◯	◯	◯
your parents advised against it.	◯	◯	◯
you have already had HPV, or an abnormal Pap, so the vaccine will not help you.	◯	◯	◯

Other (please specify)

SECTION G) last questions!

74. Would you like to be contacted about participating in a large, four-year study of thousands of young women to learn more about healthy living in women in your age range?

- ◯ Yes
- ◯ No

75. On a scale from 1 to 5:

	1 = not honest at all	2	3	4	5 = completely honest
how honest were you in your answers to the questionnaire?	◯	◯	◯	◯	◯

76. For each reason below, please fill in the circle that best represents the usefulness of a Young Women's Advisory Group (the young women who greeted you today).

(If you DID NOT visit the study site or DID NOT meet any young female peer support workers, PLEASE SKIP THIS QUESTION)

The young female staff were useful in:

	1 = not useful at all	2	3	4	5 = very useful
Providing you valuable information about health living	◯	◯	◯	◯	◯
Making you feel comfortable	◯	◯	◯	◯	◯
Answering general questions	◯	◯	◯	◯	◯
Making your visit enjoyable	◯	◯	◯	◯	◯

77. Who do you prefer to provide you with information about healthy living? For each person below, please rank your preference, from first to last choice:

	First choice	2nd choice	3rd choice	4th choice	Last choice
Young female peer support worker	◯	◯	◯	◯	◯
Nurse	◯	◯	◯	◯	◯
Doctor	◯	◯	◯	◯	◯
Research assistant	◯	◯	◯	◯	◯
Other person	◯	◯	◯	◯	◯

If "Other person", please specify

[ ]

78. How embarrassing did you find this questionnaire?

- ◯ Extremely embarrassing
- ◯ Very embarrassing
- ◯ Quite embarrassing
- ◯ Slightly embarrassing
- ◯ Not at all embarrassing

Almost finished!!

79. We would like to call you to get your feedback about this questionnaire and discuss any issues or questions that concerned you. If you do NOT want us to call you, please check the box below

◯ I DO NOT want you to call me to get feedback about this questionnaire

END OF QUESTIONNAIRE!!

That was the final question. Thank you for participating in our study.

80. Feel free to make comments below. We would especially like to hear from you about what health issues concern you the most.

Comments:

WE ENCOURAGE YOU TO CALL US (1800 188 385) AT ANY TIME WITH QUESTIONS, FEEDBACK OR CONCERNS.

Or you may contact any of the following people, depending on the nature of your query:

FOR FURTHER INFORMATION about this project or if you have any problems that may be related to your involvement in the project (for example, feelings of distress), you can contact the principal researcher, or associate researchers:

Name: Professor Suzanne Garland
Role: Director, Royal Women's Hospital Department of Microbiology and Infectious Diseases
Telephone: (03) 8345 3671

Name: Dr. Elya Moore
Role: Senior Clinical Epidemiologist, Royal Women's Hospital Department of Microbiology and Infectious Diseases
Telephone: (03) 8345 3692

Name: Dr. Yeshe Fenner
Role: Project Officer, Royal Women's Hospital Department of Microbiology and Infectious Diseases
Telephone: (03) 8345 3682

FOR COMPLAINTS:
If you have any complaints about any aspect of the project, the way it is being conducted or any questions about being a research participant in general, then you may contact the Consumer Advocate at the Royal Women's Hospital:

Name: Consumer Advocate
Telephone: (03) 8345 2290
